# Note on the Processes of Menstruation

**Published:** 1895-02

**Authors:** Lawson Tait

**Affiliations:** 7 The Crescent; Birmingham, Eng.


Buffalo Medical and Surgical Journal

Vol. XXXIV. FEBRUARY, 1895. No. 7.

### Original Communications.

### NOTE ON THE PROCESSES OF MENSTRUATION.

By LAWSON TAIT, F. R. C. S., Birmingham, Eng.

By the kindness of its author, Mr. Walter Heape, of the Cambridge Zoological Museum, who has supplied me with a copy, I am enabled to make some comments on his valuable paper published by the Royal Society, The Menstruation of Semnopithaces Entellus. In a new line of research he has obtained very considerable promise of success ; certainly he has, I think, so completely settled many of the problems that I may at once indicate that in which alone he still requires further search before he can completely satisfy us. I mean the process of denudation. Here he fails, because, I think, the tissue he has been working at is so delicate and so definitely terminated on the slide that his hardening methods are altogether faulty, and nothing will suffice (as I have argued over and over again) but the examination after staining of sections of absolutely fresh tissue cut by the freezing process.

He has made a marked advance in adopting a division of the menstrual cycle into four distinct periods, each with their distinct histological characters, indicating, on the whole, eight stages. These are :

A. Period of rest.
 Stage I.—The resting stage.

B. Period of growth.
 Stage II.—The growth of the stroma.
 Stage III.—The increase of vessels.

C. Period of degeneration.
 Stage IV.—The breaking down of vessels.
 Stage V.—The formation of lacunæ.
 Stage VI.—The rupture of lacunæ.
 Stage VII.—The formation of menstrual clot.

D. Period of recuperation.
 Stage VIII.—The recuperation stage.

At first sight, and to those who have only superficially considered these important phenomena, the above table will look like mere pedantic tautology, but careful reading of the body of the paper will soon remove such an impression. Good reasons will appear for its foundation on real fact, and it completely justifies the position I have occupied for many years—that we should not speak of menstruation occurring once a month, but we should speak of the menstrual processes occupying a whole month, for the periods of rest and recuperation (A and D) are as important parts of the process as any other—indeed for impregnation they are probably far more so, the last (D) being probably the only essential of all four.

The arrangement of the paper is so logical and complete that the author seems to leave a structure which will probably be far more lasting than most which have gone before, and it is a perfect mine of record of the astounding discrepancies in the statements of fact concerning the phenomena of menstruation. Writers of text-books who talk so glibly of the "accepted doctrine that detuscence of an ovum takes place rhythmically once a month, and is the cause of the external manifestations known as menstruation," will be astonished to find a tabular statement which shows that "the majority of recent writers are in favor of the view that ovulation is not necessarily coincident with menstruation." The question is not merely a scholastic one, not merely a question of physiology, when it is remembered that on the recurrence of menstrual flows after removal of the uterine appendages it has been asserted over and over again that a piece of ovary must have been left, as if a strangulated fragment of an organ could carry on mature function.

I have not space enough to follow Mr. Heape in many of his interesting conclusions, but he touches, by the way, on some very interesting points bearing on the great questions of the day, and shows us how far too easily we are led in phases of fashion, even in questions of physiology. One of them at the present moment is the action of leucocytes, or, as their last nomenclature gives it us, phagocytes.

Speaking of the clearing away of the hemorrhagic dèbris, Mr. Heape says, "according to Metchnikoff, the congregation of the leucocytes in the vessels near the surface indicates the presence there of noxious material. The casting off of the menstrual mucosa together with this irritating substance, and the rapid clean healing of the wounded surface would have the effect of clearing away that which the leucocytes were summoned to absorb; their protective presence in this instance, then, is not required, and they may be said to have been induced to appear on the scene to undertake duties which are otherwise performed." The delicate irony of the last few words is fun as much as can be expected in the Transactions of the Royal Society, but it bursts the "cell-policemen" theory of Metchnikoff as completely as dynamite.

Concerning the relations of ovulation with the menstrual process, I need only copy a few of Mr. Heape's words to show that the belief, so deeply treasured in the mind of the medical profession, that they are concurrent and interdependent, is a hallucination. It is many years since I proved this, with Dr. A. E. Clarke's assistance in human menstruation, but I have made no sensible impression on the minds of my brethren, certainly not on the text-books. (P. 442) "Thus, as far as discharged follicles are concerned, it is seen that out of forty-two menstruating specimens, none of which had recently borne young, only two had a recent corpus luteum in their ovaries. Such a result appears to me amply sufficient to warrant the statements:

"First, that ovulation does not necessarily occur during each menstrual period;

"Secondly, that menstruation is not brought about by ovulation;

"It is no doubt possible that an increased blood supply may affect the ovary, and may have a tendency, by means of pressure, to induce ovulation where an ovum in a sufficiently advanced stage of development is present. In my opinion this is a very probable view; it does not, however, permit of the inference that an ovum is actually detusced at each menstrual period, but rather that if an ovum in a sufficiently ripe state be present, the congestion which occurs during the menstrual period may cause it to be shed." This, of course, is the only feasible explanation of the relative infrequency of human gestation to what would be the case if impregnation *might* follow every menstrual period. Mr. Heape is further of opinion that his evidence points to the conclusion that the ripening of the ovary is independent of the process of menstruation, and that ovulation is neither the cause nor the necessary result of menstruation. As a corrollary of this, of course, the fetish of the corpus luteum comes in for wholesale destruction: "In the first place I greatly doubt the possibility of determining accurately when a Graafian vesicle is perfectly ripe; and in the second place I doubt if it is possible to be assured of the exact age of a 'false'

corpus luteum." Yet in a court of justice the corpus luteum has been the point on which life and death has depended—not so very long ago.

The importance of these views is not one of mere dialectic importance, for I have contended, and I contend still, that it is impossible for us to arrive at proper conclusions concerning the pathology of the uterine appendages until we get quit of the still accepted view that ovulation is the cause of menstruation. My opposition to this view has, more than anything else, brought upon me the opposition and misrepresentation to which my surgical doctrines have been subjected.

7 THE CRESCENT.

